# Idiosyncratic Warfarin-Induced Hepatocellular Injury: A Reversible and Uncommon Occurrence

**DOI:** 10.7759/cureus.96644

**Published:** 2025-11-12

**Authors:** Usamah Al-Anbagi, Aiat O Elmabrouk, Aia A Elimam, Baraa M Awadalla, Muhammad Sharif, Bassem Al Hariri, Abdulqadir J Nashwan, Memon Noor Illahi

**Affiliations:** 1 Internal Medicine, Hamad Medical Corporation, Doha, QAT; 2 Medical Education, Hamad Medical Corporation, Doha, QAT; 3 Internal Medicine, Hamad General Hospital, Doha, QAT; 4 Nursing and Midwifery Research, Hamad Medical Corporation, Doha, QAT

**Keywords:** case report, direct oral anticoagulants (doacs), drug-induced liver injury, hepatotoxicity, transaminitis, warfarin

## Abstract

Warfarin is a widely used oral anticoagulant for the prevention and treatment of thromboembolic events. While its safety profile is well established, hepatotoxicity is an extremely rare but potentially life-threatening adverse effect. We describe a 41-year-old man who developed acute hepatocellular injury within 24 hours of initiating warfarin for upper limb deep vein thrombosis (DVT). He had no past medical history, risk factors for liver disease, or exposure to hepatotoxic agents. Baseline liver function was normal, and alternative causes, including viral and autoimmune hepatitis, were excluded. Marked elevations in alanine aminotransferase (ALT) and aspartate aminotransferase (AST) were observed, which improved rapidly after discontinuing warfarin. The patient was transitioned to apixaban, with complete normalization of liver enzymes on follow-up. This case illustrates a rare but reversible episode of warfarin-induced hepatotoxicity. Early recognition, prompt discontinuation of the drug, and switching to a direct oral anticoagulant (DOAC) facilitated complete recovery. Clinicians should remain vigilant for hepatic injury during warfarin therapy, particularly soon after initiation, to prevent progression to severe dysfunction.

## Introduction

Warfarin, a vitamin K antagonist, is one of the most commonly prescribed oral anticoagulants for both the prophylaxis and treatment of thromboembolic conditions such as deep vein thrombosis (DVT), pulmonary embolism, and stroke in patients with atrial fibrillation or mechanical heart valves [1]. Although highly effective, warfarin has a narrow therapeutic index and is well known for its risk of bleeding complications. In contrast, warfarin-induced hepatotoxicity is exceedingly rare and often underrecognized [2,3].

Large observational studies suggest that the risk of hepatotoxicity may be higher with warfarin compared to newer oral anticoagulants (direct oral anticoagulants (DOACs)), although the absolute incidence remains low. In one cohort of atrial fibrillation patients treated with warfarin, hepatotoxicity occurred in approximately 3.4% of cases, a rate lower than that observed with DOACs [4,5]. Although the mechanism is unconfirmed, the injury is idiosyncratic. The frequent rapid recurrence of more severe hepatotoxicity upon rechallenge strongly suggests an underlying immunologic pathogenesis [2].

Clinically, acute hepatocellular injury following warfarin initiation is rare, with only a few cases reported in the literature. For example, a 64-year-old woman experienced gastrointestinal bleeding and acute liver failure during warfarin treatment, which improved following drug withdrawal and supportive measures [6].

We report the case of a 41-year-old man who developed acute hepatocellular injury within 24 hours of starting warfarin for upper limb DVT, with biochemical recovery after discontinuation. This case highlights the importance of early recognition and appropriate management of this uncommon but clinically significant adverse effect.

## Case presentation

History

A 41-year-old male with no prior medical history presented with a three-day history of progressive pain and swelling in his left upper limb, initially affecting the forearm and gradually extending to the upper arm and shoulder. He denied any history of trauma, falls, heavy lifting, or strenuous exercise. There were no recent intravenous cannulations, injections, or blood sampling procedures on the affected limb. The patient reported no recent hospitalizations, surgeries, prolonged immobilization, or long-distance travel. He denied symptoms such as chest pain, palpitations, shortness of breath, cough, or hemoptysis. There was no history of liver disease, alcohol use, smoking, or illicit drug use. He denied recent use of herbal supplements, over-the-counter medications, or exposure to known hepatotoxins. There was no history of viral hepatitis, autoimmune disease, or systemic illness, and no family history of liver disease.

Examination

On examination, the patient was alert, oriented, and afebrile. There was no congestion in the throat or nasal mucosa. No pallor, cyanosis, clubbing, jaundice, pedal edema, or significant lymphadenopathy were noted, and jugular venous pressure was normal. Examination of the left upper limb revealed diffuse swelling of the forearm (non-tender), swelling of the upper arm associated with tenderness, and swelling of the shoulder without tenderness. Prominent superficial veins were noted over the left shoulder and chest wall. Cardiovascular examination revealed a normal heart rate and rhythm, with normal S1 and S2 sounds and no murmurs. Respiratory examination showed equal bilateral air entry with no added sounds. Abdominal examination was unremarkable, with a soft, non-tender, and non-distended abdomen and no hepatosplenomegaly. Neurological examination revealed no focal deficits or meningeal signs.

Investigations and management

Initial laboratory investigations showed normal liver and renal function tests, a slightly elevated D-dimer, prolonged activated partial thromboplastin time (aPTT), normal prothrombin time (PT), and otherwise unremarkable blood work. Doppler ultrasound of the left upper limb confirmed DVT involving the proximal brachial and basilic veins. The patient was admitted with a diagnosis of left upper limb DVT and started on enoxaparin 1 mg/kg twice daily. On day 4 of admission, repeat liver function tests remained within normal limits. Warfarin therapy was started on day 5 at a dose of 7 mg daily.

**Figure 1:**
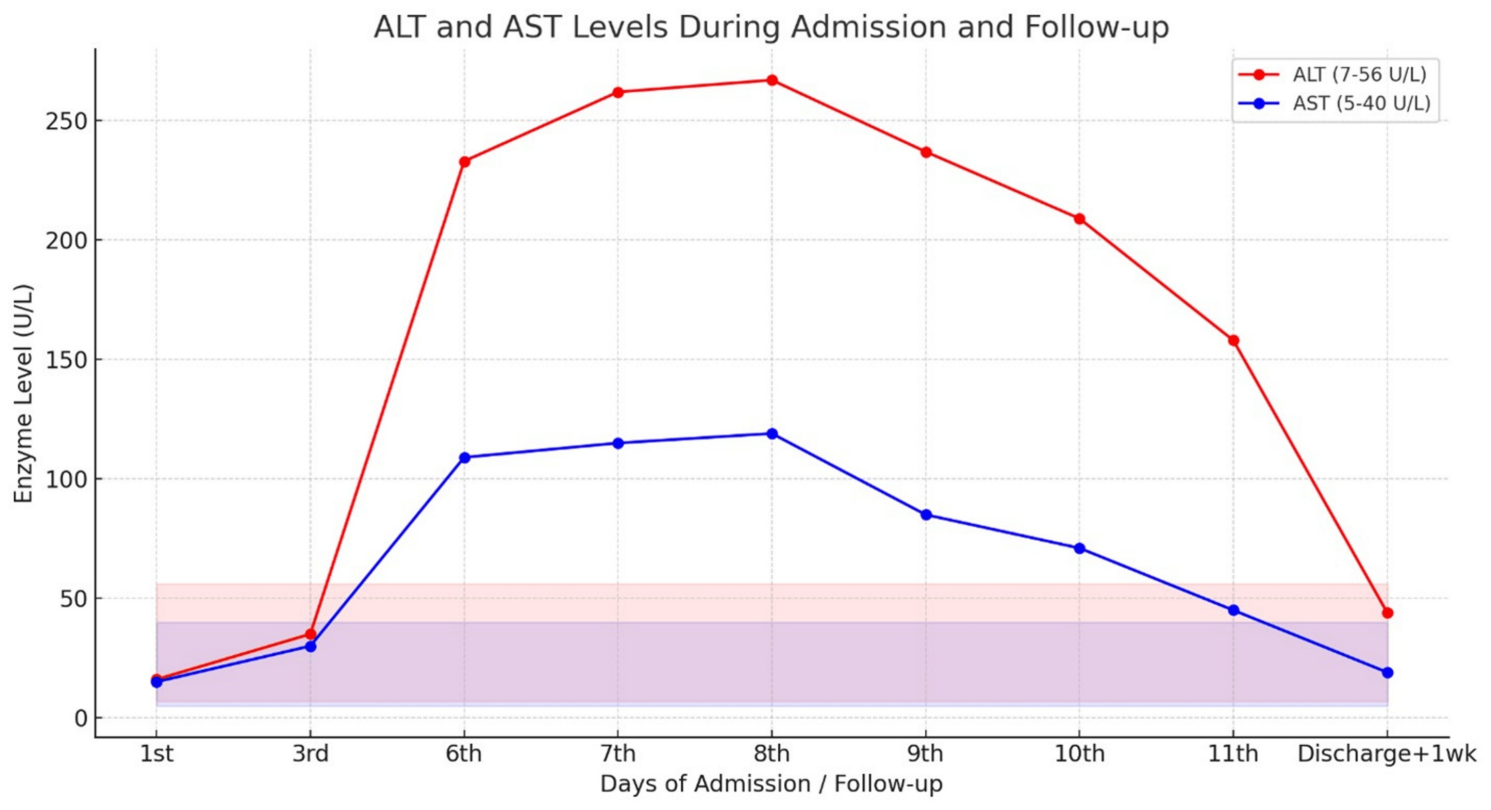
Trend of ALT and AST levels during hospital admission and post-discharge follow-up This figure illustrates the serial measurements of alanine aminotransferase (ALT) and aspartate aminotransferase (AST) levels in a patient during hospitalization and one week after discharge. ALT (red line; reference range 7–56 U/L) and AST (blue line; reference range 5–40 U/L) both increased markedly by day 6, peaking around day 8–9 before gradually declining toward near-normal values by one week post-discharge. The shaded regions represent the normal physiological ranges for each enzyme. The pattern indicates acute hepatocellular injury with subsequent recovery following clinical management and withdrawal of the suspected offending drug.

Within 24 hours of warfarin initiation, the patient exhibited an acute rise in liver enzymes, with alanine aminotransferase (ALT) increasing to more than six times baseline and aspartate aminotransferase (AST) rising to three times baseline (Figure 1). The liver enzymes continued to rise over the following days, prompting discontinuation of warfarin after three days of therapy. Liver function tests began to improve one day after cessation, corroborating the diagnosis of warfarin-induced liver injury and transaminitis. Liver enzymes gradually normalized, and the patient remained clinically stable at discharge. Anticoagulation was transitioned to apixaban 5 mg twice daily. One week post-discharge follow-up revealed complete normalization of AST and ALT values.

## Discussion

Symptoms of liver injury can range from asymptomatic elevations in liver enzymes to fulminant hepatic failure. Because of its rarity, clinicians may not readily associate new hepatic dysfunction with warfarin use. Several cases have been reported; one man developed transaminase elevations by the fourth day of therapy, which resolved upon discontinuation of warfarin [6], while another case involved a 79-year-old woman who developed cholestatic liver injury shortly after initiating warfarin for DVT, which improved upon switching to enoxaparin; however, when apixaban was subsequently started, her liver function tests worsened again, leading the authors to conclude that apixaban should be avoided in patients with a history of warfarin-induced hepatotoxicity, suggesting a potential cross-reactivity or shared idiosyncratic susceptibility between the two anticoagulants [7].

Although hepatotoxicity has been reported with both vitamin K antagonists and DOACs, the prevalence appears higher with warfarin. Registry and clinical trial data confirm that hepatotoxicity is rare with DOACs, whereas warfarin has consistently been linked to higher rates of abnormal liver function tests and clinically significant hepatocellular injury [3,8-11]. In a large randomized trial comparing rivaroxaban and warfarin for atrial fibrillation, liver enzyme elevations were more frequent in the warfarin arm, supporting the view that DOACs may be safer alternatives with respect to hepatic outcomes [9].

The pathogenesis of warfarin-induced hepatotoxicity remains incompletely understood, but several mechanisms have been proposed based on experimental and preclinical studies. Warfarin may induce hepatocyte injury through oxidative stress, suppression of the nuclear factor erythroid 2-related factor 2 (NRF2) antioxidant pathway, increased cytochrome P450 2C9 activity [12], lipid peroxidation, and iron deposition (hemosiderin accumulation), ultimately leading to hepatocellular apoptosis and acute liver injury [12]. While these findings are largely derived from experimental models, they provide a biologically plausible explanation for this rare idiosyncratic reaction.

The diagnosis of warfarin-induced liver injury depends on excluding alternative etiologies and demonstrating a strong temporal association with drug exposure. Other potential causes, including viral hepatitis, autoimmune hepatitis, ischemic injury, and hepatotoxicity from concomitant medications, must be systematically ruled out. Structured tools, such as the Roussel Uclaf Causality Assessment Method (RUCAM), can help objectively assess the likelihood of a drug-induced reaction. In our patient, the RUCAM score was 8, indicating probable causality for warfarin-induced hepatocellular injury [9].

Management is primarily supportive and depends on prompt discontinuation of the offending agent. Re-challenge with warfarin is contraindicated due to the risk of recurrence and the possibility of a more severe reaction. In our patient, switching to apixaban provided effective anticoagulation and avoided further hepatic injury, consistent with emerging evidence supporting DOACs as safer alternatives in patients with or at risk of liver disease [7,9]. While routine liver function monitoring is not generally recommended during warfarin therapy, it may be warranted in patients who develop symptoms of hepatic dysfunction or in those with preexisting liver disease.

Early recognition is crucial, as timely intervention can prevent progression to acute liver failure, which has been reported in rare cases. Most patients recover with supportive care alone, such as vitamin K administration in the setting of coagulopathy. Severe cases may require intensive monitoring and additional interventions, including management of hepatic encephalopathy. In our case, the patient improved with conservative measures alone [9].

This case adds to the limited literature on warfarin-induced hepatotoxicity and underscores the importance of clinical vigilance when initiating anticoagulation. While warfarin remains a cornerstone therapy, awareness of its rare hepatic adverse effects is essential. Warfarin-induced hepatotoxicity should be considered in the differential diagnosis of acute liver injury, as early recognition and drug withdrawal usually result in full recovery. Clinicians must balance the benefits of anticoagulation with its potential hepatic risks and consider safer alternatives when available. Further research into the mechanisms and predictive markers of this adverse effect is warranted to guide prevention and management.

## Conclusions

Warfarin-induced hepatotoxicity is a rare but clinically significant adverse effect that should be considered in patients who develop acute liver injury during anticoagulation therapy. Early recognition and prompt discontinuation of warfarin are essential, as they usually result in complete recovery. Clinicians should weigh the therapeutic benefits of warfarin against its potential hepatic risks and, when appropriate, consider transitioning to DOACs as safer alternatives.
